# Drivers of university–business cooperation of university faculty from the social cognitive theory perspective

**DOI:** 10.3389/fpsyg.2022.1013774

**Published:** 2022-10-11

**Authors:** Hongwei Zhang, Xiyue Chen, Yang Lv, Mengru Li

**Affiliations:** ^1^School of Economics, Sichuan University, Chengdu, China; ^2^School of Public Administration, Sichuan University, Chengdu, China; ^3^Office of Academic Affairs, Chengdu University, Chengdu, China; ^4^College of Teachers, Chengdu University, Chengdu, China

**Keywords:** university-business cooperation, drivers, university faculty, motivations, internal mechanism, external environment

## Abstract

As an independent research field, there is growing attention to university–business cooperation (UBC). However, few studies focus on the driving factors of UBC, which remains an open problem in this area. This study analyzes a broad mix of drivers underlying seven UBC activities, namely, curriculum development and design (CDD), student mobility (SD), lifelong learning (LLL), professional mobility (PM), research and development (R&D), commercialization (COM), and entrepreneurship (ENT), and discusses the internal mechanism and external environment of higher education institutions (HEIs) as the moderator variable affecting UBC activities and individual motivations. Specifically, based on the social cognition theory, the independent variables include motivations (money, career, research, education, and social), the internal mechanism (support mechanism, strategic mechanism, and management mechanism), and the external environment (policy environment, economic environment, and cultural environment) are designed. The aforementioned seven UBC activities are taken as dependent variables. This work takes university faculty as the research object. Through empirical analysis, it demonstrates that the combination of driving factors of different UBC activities has its particularity. Furthermore, the results showed that the internal mechanism and external environment of HEIs could positively moderate the relationship between individual motivations and UBC activities. In terms of theoretical contribution, this study reveals the combination of factors that drive university faculty to engage in UBC. On the other hand, it can provide a reference for policymakers and managers to better development of UBC.

## Introduction

University–business cooperation (UBC) is a common topic in industrial economics and school education. It can promote social innovation through knowledge and technology transfer. Therefore, it is considered an essential economic and social development source. Generally speaking, UBC is understood as the interaction between higher education institutions (HEIs) and businesses for mutual benefit (Bellini et al., 2019). Similar concepts involve university–firm cooperation and cooperative education (Rosivalda and Mário, 2022).

Based on the significance of UBC and its widespread existence in countries worldwide, researchers regard it as a new field and have contributed many new concepts and discoveries. Although much research has been carried out on UBC, most studies have only been restricted to the mode of research and development (R&D), commercialization, and related issues (Santos et al., 2021). Even though several researchers have already realized that UBC has distinctive value in HEIs achieving its missions, UBC in HEIs is not fully explained (Forsyth et al., 2009; Orazbayeva et al., 2019a,b), which remains an open problem.

To fully understand UBC, we need to explore the factors that drive individuals to carry out UBC activities. Although a few authors have paid attention to this issue, these studies still lack a comprehensive perspective. For example, some authors studied the individual motivation of academics to engage in cooperative research and commercialization (Lam, 2007; D'Este and Perkmann, 2011), while others analyzed the motivation factors of European university academics to engage in education-driven UBC activities (Orazbayeva et al., 2019a). It can be seen that researchers often only focus on one aspect of UBC activities.

On the other hand, to understand UBC from the standpoint of HEIs, it should include behaviors from two types of participants: academics and administrative staff (Bstieler et al., 2017). The motivation of university administrative staff to engage in UBC also affects the final effect of UBC activities to a great extent, which is always ignored. In addition, researchers often separate individual motivation from environmental factors. As far as we know, no has been attention paid to the moderate effect of environmental factors on individual motivations.

This study addresses these shortcomings by exploring the drivers underlying the UBC activities in HEIs. Moreover, UBC is taken place in three domains, namely, education, research, and valorization, and refers to seven activities. In the education domain, three activities are involved: (a) curriculum development and design (CDD), (b) student mobility (SD), and (c) lifelong learning (LLL). The research domain comprises (d) professional mobility (PM) and (e) research and development (R&D). (f) Commercialization (COM) and (g) entrepreneurship (ENT) are involved in the valorization domain. Furthermore, many factors affect different activities (Lam, 2007). In order to deepen the understanding of UBC, this study explores the drivers of university academics and administrative staff when engaging in UBC activities. This study applies a social cognitive theory perspective, which emphasizes the impact of environmental factors on individual motivation and behavior; drawing on the social cognitive theory, this study includes three driving factors: individual motivation, internal mechanism, and external environment.

Precisely, the methods of questionnaire investigation and quantitative data analysis are used to discover the driving factors of university faculty engaged in different UBC activities and the regulatory mechanism of environmental factors on individual motivations. Specifically, based on data collection, UBC activities are taken as dependent variables, and drivers as independent variables to analyze the correlation between drivers and UBC activities. In the multivariable analysis of the general linear model, interaction models are constructed to analyze the regulating role of the internal mechanism and the external environment as moderator variables between personal motivation and UBC activities. In general, this article offers an essential contribution to the understanding of UBC in HEIs. Drawing on a questionnaire, it outlines a complicated motivation combination of university faculty engaging in UBC, thus going beyond the previous research focusing only on commercialization and related issues. Furthermore, it provides a more comprehensive method and new viewpoint for UBC research and also offers some valuable information for promoting UBC activities in practice.

## Theoretical background

### University-business cooperation

From the viewpoint of HEIs, the emphasis on UBC comes from reducing the skill gap and improving students' employability, including curriculum construction, student mobility, and other activities. In recent years, more and more attention has been paid to fulfilling the responsibility of serving society through UBC. Its specific form is often to cultivate all talents needed for regional economics and society development, solve scientific and technological problems for industry or enterprises, and provide complementary services for regional economic development.

Therefore, professional mobility, research and development, commercialization, entrepreneurship, and other activities are widespread at UBC. UBC can enrich the knowledge structure, improve teaching skills, develop new research directions, and obtain monetary resources simultaneously for university faculty. It is generally believed that UBC fields include consultation, curriculum, training, exchange of views, internships, participation in meetings, personnel exchange, and others (Czerwinska-Lubszczyk et al., 2020).

While the current information is that UBC is often driven by external profit motivation (Bozeman and Boardman, 2013), these findings are put forward in research on commercialization, patents, and licenses. From another point of view, research on education-related activities shows that extrinsic motivation does not positively drive people to engage in UBC activities (Geuna and Rossi, 2011). It can be seen that there is a significant gap in motivation combinations under different circumstances. Academics often initiate UBC in Europe through personal relations, sporadically and informally (Orazbayeva et al., 2019a). UBC has national and local policy guidance and relatively complete institutional support in China. Generally speaking, UBC activities with particular influence in China are initiated through formal channels, with the participation of officials, and subject to government supervision and help. Therefore, the existing research on the motivation of UBC is not applicable to explain the situation of UBC in China.

### UBC activities

In this article, UBC is defined as the formal interaction and collaboration between HEIs and members of any public or private external organization to achieve the transfer or exchange of knowledge, technology, or other properties. Although significant, informal interactions, such as conferences and expositions, are not considered activities of UBC but precursors of cooperation (Geuna and Rossi, 2011). Existing studies on the UBC subject have been mostly restricted to only one aspect (Lee, 1996; Zucker and Darby, 1996). Although activities such as R&D or technology transformation are well known to the public, they still cannot represent the overall picture of UBC activities. Moreover, UBC activities are interrelated and interactive (Zucker and Darby, 1996). Focusing only on one aspect of activities, irrespective of the aspect, is not enough for understanding UBC. On the other hand, even in HEIs, research- and valorization-driven UBC activities have the same importance as education-driven activities in China.

Therefore, this study adopts a broader perspective to understand UBC, unlike previous studies focusing only on the educational domain. Thus, in our work, UBC activities include seven activities in the three aspects mentioned earlier. This study is based on seven types of UBC activities and takes UBC activities as the dependent variable of quantitative analysis. In order to carry out the research better, it is necessary to clarify the specific meaning of each type of UBC activity. Specifically, the activities in the education domain are as follows:

(a) Curriculum development and design (CDD): Here, personnel from external institutions participating in the curriculum design and construction, or enterprise personnel providing lectures to HEI students to bring enterprise knowledge into the curriculum.
(b) Lifelong learning (LLL): HEIs provide adult education, lifelong education, or continuing education to external institutions' personnel.
(c) Student mobility (SD): It is the temporary mobility of students from HEIs to business through internships and other ways.
  UBC activities related to the research domain include the following activities:
(d) Professional mobility (PM), that is, employees exchange between HEIs and enterprises.
(e) Research and development (R&D), including joint R&D projects, scientific and technological innovation, and achievement transformation.
  The valorization domain includes the following activities:
(f) Commercialization (COM): scientific research that enters the market through intellectual property transactions.
(g) Entrepreneurship (ENT): college student or scholar entrepreneurship.

## Conceptual framework of UBC drivers

### Social cognitive theory

UBC is a complex and diverse cooperative behavior that always occurs in social interaction situations (Perkmann and Walsh, 2007). It is far from a linear knowledge transfer process. The social cognitive theory believes that individual, environment, and behavior are three parts that are independent of each other and also interact at the same time (Locke, 1987), the three are mutually causal, and every two parts have an interactive relationship (Anwar et al., 2019; Johnston et al., 2019).

Based on the understanding of UBC driving factors from the social cognitive theory, individual motivations and environmental factors are the main reasons for the occurrence and maintenance of UBC activities. On the one hand, individual motivation and some external factors drive and guide behavior. On the other hand, environmental factors such as reward and punishment mechanisms, policies, and economics will affect the continuity of individuals' behavior. From the standpoint of the HEIs, university faculty are driven by individual motivations and affected by a series of support mechanisms for UBC activities within the university, as well as the political, economic, cultural, and other factors of the society. Based on this, this study believes that the factors driving UBC activities should include three aspects and their interactions: individual motivations, internal mechanisms, and the external environment.

### Individual motivations

*Continuum* is a psychological concept that integrates intrinsic and extrinsic motivations (Ryan and Deci, 2020). Intrinsic motivation is the typical expression of human nature that has assumed a positive integration tendency, which refers to the action caused by the interest and enjoyment of the activity itself or the pursuit of its value. Extrinsic motivation is usually defined as instrumental motivation involving all behaviors that can be separated from the activity itself. It refers to an individual engaging in a specific activity or behavior to pursue a particular reward or avoid punishment. Motivation is a state of lack of behavioral intention. Individuals feel unable to complete an activity or do not believe in the expected results (Ryan and Deci, 2020).

Based on the existing research, the motivation of UBC includes five motivational orientations: money, career, research, education, and social (Linke et al., 2010; Orazbayeva et al., 2019a). Monetary orientation means people can obtain funding and financial support by participating in UBC. Career orientation means that engagement in UBC can increase the opportunity for promotion or enhance the popularity in the university. Research orientation refers to putting research into practice through UBC activities or obtaining new research perspectives through UBC activities. Educational orientation means improving teaching ability or students' employability through UBC. Social orientation aims to serve society and promote development through UBC activities.

**Figure 1:**
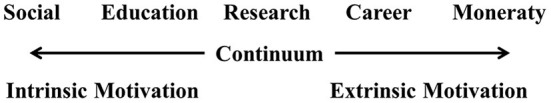
Continuum.

According to the definition of the continuum, it can be classified into extrinsic motivation and intrinsic motivation. Money and career orientations can be regarded as at the end of extrinsic motivation, while career, research, and society are at the end of intrinsic motivation. It should be noted that there is no clear boundary between them, but they are continuously distributed from the outer end to the inward end of the motivation continuum, as shown in Figure 1.

There is controversy about whether money can effectively motivate academics to engage in UBC activities. A European survey shows that money and career orientation motivations do not significantly correlate when academics participate in education-driven UBC activities. While some studies believe that monetary rewards are mainly related to the scientific activities of academic personnel, which are paid in the form of salary increases or research funds according to the results achieved or commercialized returns (Van Norman and Eisenkot, 2017). Since the seven UBC activities involved in this study include the education domain, and the research and valorization domain, this study assumes that monetary and career motivations can affect UBC activities to a certain extent.

H1: Monetary orientation motivation positively affects UBC activities.
H2: Career orientation motivation positively affects UBC activities.
H3: Research orientation motivation positively affects UBC activities.
H4: Education orientation motivation positively affects UBC activities.
H5: Social orientation motivation positively affects UBC activities.

### Internal mechanism and external environment

According to the theory of social cognition, understanding faculty engagement in UBC activities from the standpoint of HEIs involves three aspects: individual motivations, internal mechanisms, and the external environment of HEIs. The internal mechanism and external environment not only are the driving factors of engaging in UBC activities but also moderate UBC activities by affecting individual motivation, so they are also the moderator variables between motivation and UBC activities.

The internal mechanism is the measure formulated by the HEIs on the development and management of UBC to create favorable conditions for UBC to achieve better UBC goals (Bovey and Hede, 2001). The development and promotion of UBC are hindered by many obstacles, such as difficulties in finding suitable partners and different motivations/values between HEIs and businesses (Czerwinska-Lubszczyk et al., 2020). If there is no internal mechanism, UBC is likely to become a short-term, unsustainable activity that depends on individual motivation and is arbitrary.

This study believes that the internal mechanism includes three aspects, namely, the support mechanism, strategic mechanism, and management mechanism. The support mechanism is a series of mechanisms set up by HEIs to provide favorable conditions for UBC, including salary, evaluation and employment, rewards, and punishments mechanisms, in order to create an effective and consistent mechanism for appropriate support, incentive, and drive, and for obstacles from top to bottom or bottom to top. Strategic mechanism refers to the long-term decisions about UBC drafted and implemented by HEIs, so they can be realized and decided. Generally, it includes document strategy mechanism and implementation strategy mechanism. UBC can be created within the university by formulating a strategic development mechanism to promote UBC development better. Management mechanism refers to the actions taken by HEIs to support and develop UBC, including communication, link, training, and others.

The literature shows that the HEI operation environment will also impact UBC development (Bruneel et al., 2010). As a cooperative activity in education and business, UBC will be affected by political, economic, cultural, legal, and historical factors. The complex mixture of these factors forms an environment that can support or inhibit UBC (Alunurm et al., 2020). This study believes that UBC activities are affected by three external environmental factors: the policy environment, which involves the laws, policies, and official behavior orientation of UBC activities; economic environment, which means the economic situation of the region where UBC activities are carried out; and cultural environment, which refers to the social culture, history, and preferences related to UBC in which the HEI is located.

H6: Support mechanism positively affect UBC activities.
H7: Support mechanism as a moderator variable positively affect UBC activities.
H8: Strategic mechanism positively affect UBC activities.
H9: Strategic mechanism as a moderator variable positively affect UBC activities.
H10: Management mechanism positively affect UBC activities.
H11: Management mechanism as a moderator variable positively affect UBC activities.
H12: Policy environment positively affect UBC activities.
H13: Policy environment as a moderator variable positively affect UBC activities.
H14: Cultural environment positively affect UBC activities.
H15: Cultural environment as a moderator variable positively affect UBC activities.
H16: Economic environment positively affect UBC activities.
H17: Economic environment as a moderator variable positively affect UBC activities.

## Methodology

This study intends to use quantitative analysis to explore the relationship between driving factors and UBC activities. First, it defines UBC activities as the dependent variable. There are seven dependent variables corresponding to the seven activities of UBC. In addition, the driving factors are independent variables, including three aspects, thus a total of eleven variables, which correspond to the individual motivations, internal mechanism, and external environment described earlier. The results are based on the correlation analysis and multivariate analysis of the general linear model. The specific content is shown in the data analysis section.

As for the method of collecting data, this study carried out an online survey. This questionnaire was distributed to 10 public universities in southwest China from March to May 2022, requiring academics and administrative staff who have engaged in UBC activities to fill in. A total of 462 questionnaires were finally collected in this study, of which 399 were valid, and the effective response rate was 86.4%. Among them, there were 282 academics and 117 staff; 187 men and 212 women; and 32 were 30 years old, 136 were 30–35 years old, 108 were 35–40 years old, 90 were 40–50 years old, and 33 were older than 50 years.

The questionnaire includes two parts. The first part is the basic information of the respondents, including their school, identity, age, gender, academic background, and experience of engagement in UBC activities. The second part is the main body of the questionnaire, including 18 questions. In order to measure the degree of respondents' engagement in UBC activities, seven dependent variables were designed in the survey. Each variable corresponds to a topic, thereby corresponding to the seven UBC activities involved in this study, namely, (a) curriculum development and design (CDD), (b) student mobility (SD), (c) lifelong learning (LLL), (d) professional mobility (PM), (e) research and development (R&D), (f) commercialization (COM), and (g) entrepreneurship (ENT). Respondents were given a 10-point Likert scale (1 = not at all, 10 = very high) to indicate their level of engagement in UBC activities mentioned in the questionnaire. The expression adopted in the questionnaire is as follows: please select appropriate numbers from 1 to 10 to indicate your participation in the following activities, 1 = not at all, 10 = very high.

In order to measure the influencing factors of respondents' participation in UBC activities, 11 questions were designed in the questionnaire, corresponding to the 11 independent variables in this study, covering three dimensions: motivation, internal mechanism, and external environment. The motivation dimension includes monetary orientation, career orientation, research orientation, educational orientation, and social orientation. The internal mechanism dimension includes the support, strategic, and management mechanisms. The external environment includes the economic, policy, and cultural environment. The expression adopted measures the extent to which the following factors motivate individuals to engage in UBC activities (1 = not at all, 10 = very high).

## Data analysis

In order to examine whether the survey results are reliable and practical, this study analyzes the reliability and validity of the questionnaire results. Reliability refers to the consistency or stability of the measurement results, that is, whether the measurement tool can stably measure the items (Mendoza et al., 2000). This study uses Cronbach's α coefficient to measure the consistency within the questionnaire (Tisak and Tisak, 1996). Validity is the degree to which measuring tools or means can accurately measure things, which is reflected by the KMO value in this study (Tisak and Tisak, 1996). Good reliability is the basis of validity. The α coefficient in this study is 0.721, and the KMO value is 0.728. According to the commonly used measurement standards, the reliability and validity are within the appropriate range, which indicates that the data of the text have good reliability and can reflect the problem to be studied to an acceptable extent.

Common method bias (CMB) refers to the artificial covariance between the predictor variable and the target variable caused by the same data source or rater, the same measurement environment, the project context, and the characteristics of the project itself (Podsakoff et al., 2003). As this study adopted an online questionnaire survey to collect data, the procedure control cannot eliminate the deviation (Zhou and Long, 2020), and there is the possibility of CMB (Podsakoff et al., 2012). In this study, Harman's single factor test was used to detect the presence of CMB. The results show that the variance interpretation rate of the first factor is 22.009%, and the total variance is < 40%. Therefore, it can be considered that there is no significant CMB (Harris and Mossholder, 1996).

On this basis, IBM SPSS 23 was used to analyze the data. First, bivariate correlation analysis was adopted using UBC activities as dependent variables, and driving factors as independent variables. The Pearson correlation coefficient is used to express the correlation. The results are shown in Table 1. The values in Table 1 are correlation coefficients, and ^**^ means the correlation is significant at the 0.01 level (two-tailed), and ^*^ means the correlation is significant at the 0.05 level (two-tailed). There was a significant correlation between driving factors and UBC activities. In this study, if there is a significant positive correlation between the driving factors and UBC activities, it is considered that the hypothesis has been verified.

**Table 1:** Results of correlation analysis.

**Drivers**		**CDD**	**SD**	**LLL**	**PM**	**R&D**	**COM**	**ENT**
Individual motivations	Monetary	0.040	−0.163**	0.026	0.410**	0.283**	0.385**	0.178**
	Career	0.019	−0.174**	0.031	0.408**	0.259**	0.384**	0.160**
	Research	0.128*	−0.233**	0.014	0.419**	0.244**	0.395**	0.196**
	Educational	0.340**	0.295**	−0.205**	0.058	0.383**	0.065	0.224**
	Social	0.254**	0.187**	0.036	0.056	0.213**	0.095	0.255**
Internal mechanism	Support mechanism	0.207**	0.064	−0.109*	0.163**	0.146**	0.167**	0.257**
	Strategic mechanism	0.071	0.106*	−0.032	−0.086	0.055	−0.058	0.080
	Management mechanism	0.260**	0.328**	−0.259**	−0.142**	0.321**	−0.150**	0.028
External environment	Policy environment	0.193**	−0.070	−0.107*	0.440**	0.419**	0.419**	0.258**
	Cultural environment	0.039	0.087	−0.008	0.047	0.157**	0.048	0.101*
	Economic environment	0.091	−0.041	0.076	0.174**	0.051	0.156**	0.196**

*Correlation is significant at the 0.01 level (two-tailed). **Correlation is significant at the 0.01 level (two-tailed).

Furthermore, to measure the moderator effects of internal mechanisms and external environment as moderator variables on motivations and UBC activities, this study also adopted generalized linear regression analysis to test the correlation between them. The multivariable analysis of the general linear model was carried out with UBC activities as dependent variables, drivers as covariates, and individual motivation with internal mechanisms as one-to-one correspondence to establish interaction models. Similarly, one-to-one interaction models were established between individual motivation and external mechanisms. The results are shown in Table 2. According to the *F*- and *P*-values, the data were confirmed to meet the model assumptions. If the *P*-value is < 0.05, it is considered that this factor has a positive moderator effect on specific motivation with UBC activities, and the hypotheses were verified. The smaller the *P*-value, the more significant the moderator effect. Internal mechanisms and the external environment partially regulate the relationship between personal motivation and engagement in UBC activities. Moreover, the summary of the hypotheses is given in Table 3.

**Table 2:** Results of GLZM (regulatory effects of moderator variables).

	**CDD**		**SD**		**LLL**		**PM**		**R&D**		**COM**		**ENT**	
	**F**	**P**	**F**	**P**	**F**	**P**	**F**	**P**	**F**	**P**	**F**	**P**	**F**	**P**
Monetary*Support mechanism	1.925	0.022	1.052	0.441	1.134	0.351	2.085	0.012	0.993	0.514	2.591	0.002	1.793	0.037
Monetary*Strategic mechanism	1.703	0.053	0.843	0.703	0.934	0.586	1.156	0.331	0.923	0.601	1.443	0.133	2.328	0.006
Monetary*Management mechanism	2.288	0.006	1.472	0.118	0.999	0.505	1.900	0.025	1.919	0.024	2.506	0.003	1.502	0.107
Monetary*Policy environment	2.290	0.006	1.381	0.159	1.060	0.432	2.188	0.008	1.637	0.064	2.542	0.002	2.352	0.005
Monetary*Cultural environment	1.793	0.038	0.756	0.809	1.102	0.385	1.871	0.029	0.885	0.650	2.065	0.014	1.367	0.170
Monetary*Economic environment	1.624	0.068	1.313	0.201	1.143	0.342	2.344	0.005	1.153	0.332	2.485	0.003	1.599	0.074
Career*Support mechanism	1.473	0.128	1.075	0.422	1.024	0.481	1.953	0.026	1.039	0.463	2.541	0.004	1.408	0.158
Career*Strategic mechanism	1.075	0.422	0.938	0.582	0.738	0.823	1.250	0.260	0.828	0.721	1.583	0.092	1.836	0.040
Career*Management mechanism	1.526	0.110	1.480	0.127	0.912	0.617	1.469	0.132	1.345	0.195	2.182	0.013	1.212	0.290
Career*Policy environment	1.801	0.042	1.290	0.228	0.951	0.571	2.357	0.007	1.399	0.162	2.727	0.002	1.796	0.043
Career*Cultural Environment	1.423	0.152	0.979	0.534	0.997	0.511	1.663	0.070	0.708	0.857	2.026	0.021	1.300	0.223
Career*Economic environment	1.309	0.214	1.419	0.151	1.014	0.493	2.132	0.013	0.783	0.783	2.489	0.004	1.327	0.203
Research*Support mechanism	2.040	0.010	1.725	0.038	0.606	0.949	0.951	0.566	1.390	0.141	0.996	0.506	1.429	0.122
Research*Strategic mechanism	1.788	0.031	1.642	0.055	0.686	0.886	0.613	0.941	0.760	0.810	0.685	0.887	1.098	0.381
Research*Management mechanism	2.225	0.006	1.978	0.016	0.997	0.500	1.081	0.401	1.514	0.096	1.204	0.278	1.336	0.181
Research*Policy environment	2.731	0.001	1.390	0.144	0.843	0.708	0.675	0.896	1.223	0.257	0.821	0.736	1.005	0.493
Research*Cultural environment	1.575	0.073	1.553	0.079	0.683	0.887	0.905	0.624	1.111	0.367	0.859	0.685	1.012	0.484
Research*Economic environment	1.707	0.042	1.450	0.114	0.553	0.972	0.677	0.898	0.696	0.881	0.677	0.897	1.417	0.129
Educational*Support Mechanism	1.127	0.313	1.797	0.010	1.244	0.192	1.524	0.048	1.236	0.199	1.798	0.010	2.002	0.003
Educational*Strategic mechanism	1.530	0.054	1.058	0.405	1.102	0.350	1.149	0.294	1.251	0.196	1.383	0.109	0.824	0.746
Educational*Management mechanism	1.569	0.048	1.305	0.160	1.273	0.184	1.697	0.025	1.542	0.054	2.158	0.002	1.537	0.056
Educational*Policy environment	1.969	0.004	2.017	0.003	0.754	0.849	2.224	0.001	1.590	0.034	2.999	0.000	1.480	0.062
Educational*Cultural environment	1.446	0.077	1.423	0.086	1.183	0.255	1.339	0.129	1.400	0.097	1.211	0.227	1.413	0.091
Educational*Economic environment	0.949	0.571	1.791	0.010	0.907	0.637	0.987	0.510	1.150	0.286	1.049	0.417	1.820	0.009
Social*Support mechanism	1.689	0.031	1.564	0.056	0.976	0.524	1.170	0.284	0.703	0.879	1.234	0.225	1.502	0.074
Social*Strategic mechanism	0.996	0.496	1.110	0.349	0.971	0.531	1.163	0.290	0.460	0.995	0.938	0.578	0.887	0.652
Social*Management mechanism	1.769	0.021	1.113	0.345	1.479	0.080	1.098	0.363	0.896	0.639	0.963	0.542	1.176	0.277
Social*Policy environment	1.193	0.252	0.877	0.679	0.837	0.738	2.060	0.004	1.227	0.220	1.726	0.021	0.953	0.564
Social*Cultural environment	1.793	0.018	1.103	0.473	0.888	0.651	1.152	0.303	0.569	0.970	0.727	0.857	1.002	0.487
Social*Economic environment	1.976	0.007	1.092	0.370	0.677	0.908	0.908	0.624	0.595	0.961	0.859	0.695	1.049	0.425

**Table 3:** Summary of the hypotheses.

**Drivers**	**Hypotheses**	**CDD**	**SD**	**LLL**	**PM**	**R&D**	**COM**	**ENT**
Monetary	H1: monetary orientation motivation is positive affects UBC activities	False	False	False	True	True	True	True
Career	H2: career orientation motivation is positive affects UBC activities	False	False	False	True	True	True	True
Research	H3: research orientation motivation is positive affects UBC activities	True	False	False	True	True	True	True
Educational	H4: education orientation motivation is positive affects UBC activities	True	True	False	False	True	False	True
Social	H5: social orientation motivation is positive affects UBC activities	True	True	False	False	True	False	True
Support Mechanism	H6: Support Mechanism is positively affects UBC activities	True	False	False	True	True	True	True
	H7: Support Mechanism as moderator variable is positively affects UBC activities	True (partial)	True (partial)	False	True (partial)	False	True (partial)	True (partial)
Strategic mechanism	H8: Strategic mechanism is positively affects UBC activities	False	True	False	False	False	False	False
	H9: Strategic mechanism as moderator variable is positively affects UBC activities	True (partial)	False	False	False	False	False	True (partial)
Management Mechanism	H10: Management Mechanism is positively affect UBC activities	True	True	False	False	True	False	False
	H11: Management Mechanism as moderator variable is positively affects UBC activities	True (partial)	True (partial)	False	True (partial)	True (partial)	True (partial)	False
Policy Environment	H12: Policy Environment is positively affects UBC activities	True	False	False	True	True	True	True
	H13: Policy Environment as moderator variable is positively affects UBC activities	True (partial)	True (partial)	False	True (partial)	True (partial)	True (partial)	True (partial)
Cultural Environment	H14: Cultural Environment is positively affects UBC activities	False	False	False	False	True	False	True
	H15: Cultural Environment as moderator variable is positively affects UBC activities	True (partial)	False	False	True (partial)	False	True (partial)	False
Economic Environment	H16: Economic Environment is positively affects UBC activities	False	False	False	True	False	True	True
	H17: Economic Environment as moderator variable is positively affects UBC activities	True (partial)	True (partial)	False	True (partial)	False	True (partial)	True (partial)

“Partial” means that the moderator effect of environmental factors (refers to internal mechanisms and external environment) exists in motivations and UBC activities partially. Refer to the Results section for details.

## Results

According to the results of correlation analysis (shown in Table 1), it can be seen that different factors have different effects on UBC activities, so it is of great significance to investigate the correlation between individual motivations for UBC activities and the environment. Specifically, educational and social-oriented motivations, which are at the end of intrinsic motivation, are strongly positively correlated with UBC activities of CDD (0.340^**^, 0.254^**^) and SD (0.295^**^, 0.187^**^), and research-oriented motivations are significantly correlated with CDD (0.128^*^), PM (0.419^**^), R&D (0.244^**^), COM (0.395^**^), and ENT (0.196^**^). Among monetary- and career-oriented motivations, which are considered at the end of extrinsic motivation, there is no significant positive correlation with UBC activities of education-driven orientation. However, there is a significant positive correlation with PM (0.410^**^, 0.408^**^), R&D (0.283^**^, 0.259^**^), COM (0.385^**^, 0.384^**^), and ENT (0.178^**^, 0.160^**^). It is worth mentioning that monetary and career-oriented motivations have the same results in significantly positively affecting UBC activities. Similarly, educationally and socially oriented motivations have also maintained relatively consistent results. The aforementioned results show that the motivation of the outer side is more related to the activities in the valorization domain, while the motivation of the inner side is more related to the activities in education-driven motivation.

Among the three elements of internal mechanism, the support mechanism is positively and significantly related to CDD (0.207^**^), PM (0.163^**^), R&D (0.146^**^), COM (0.167^**^), and ENT (0.257^**^). The management mechanism is positively significantly related to CDD (0.260^**^), SD (0.328^**^), and R&D (0.321^**^). The strategic mechanism only shows a significant correlation with SD (0.106^*^). Among the three elements of the external environment, the policy environment strongly correlates positively with various UBC activities. The policy environment shows a significant positive correlation with CDD (0.193^**^), PM (0.440^**^), R&D (0.419^**^), COM (0.419^**^), and ENT (0.258^**^), while the economic environment shows a significant positive correlation with PM (0.174^**^), COM (0.156^**^), and ENT (0.196^**^), and the cultural environment shows a significant positive correlation with R&D (0.157^**^) and ENT (0.101^*^).

It is worth mentioning that other activities showed different degrees of correlation, except LLL. LLL has no significant correlation with all factors. Even more, there has negative significant correlation with education-oriented motivation (−0.205^**^), support mechanism (−0.109^*^), management mechanism (−0.259^**^) and policy environment (−0.107^*^). At the same time, there is a significant negative correlation between money orientation (−0.163^**^), career orientation (−0.174^**^), research orientation (−0.233^**^) with SD. The management mechanism harms PM (−0.142^**^) and COM (−0.150^**^).

As for the moderator effects of internal mechanisms and the external environment, the results are shown in Table 2. The support mechanism significantly positively moderates the relationship between motivation and UBC activities among CDD (*P*-value of moderator effects of the support mechanism between monetary and CDD mechanism is 0.022, with monetary 0.022, same as mentioned later) (monetary 0.022, research 0.010, social 0.031), SD (research 0.038, educational 0.010), PM (monetary 0.012, career 0.026, educational 0.048), COM (monetary 0.002, career 0.004, educational 0.010), and ENT (monetary 0.037, educational 0.003). The management mechanism significantly positively regulates the relationship between motivations and UBC activities among CDD (monetary 0.006, research 0.006, educational 0.048, social 0.021), SD (research 0.016), PM (monetary 0.025, educational 0.025), R&D (monetary 0.024), and COM (monetary 0.003, career 0.013, educational 0.002). The economic environment shows a strong positive relationship between CDD (research 0.042, social 0.007), SD (educational 0.010), PM (monetary 0.005, career 0.013), COM (monetary 0.003, career 0.004), ENT (educational 0.009), and motivations. The cultural environment shows a strong positive relationship with motivation in CDD (monetary 0.038, social 0.018), PM (monetary 0.029), and COM (monetary 0.014, career 0.021). The policy environment has a significant positive moderator effect on motivation among CDD (monetary 0.006, career 0.042, research 0.001, educational 0.004), SD (educational 0.003), PM (monetary 0.008, career 0.007, educational 0.001, social 0.004), R&D (educational 0.034), COM (monetary 0.002, career 0.002, educational 0.000, social 0.021), and ENT (monetary 0.005, career 0.043). However, strategic development only shows significant positive regulation between CDD and research (0.031), ENT with monetary (0.006), and career (0.040). In addition, in UBC activity, all moderator variables failed to regulate the relationship between LLL and motivations positively. The significant positive regulation of moderator variables on UBC and motivation is mainly concentrated in the three activities: CDD, PM, and COM. Among these, the regulation of monetary-oriented and CDD, monetary-oriented and PM, monetary-oriented and COM, career-oriented and COM, and research-oriented and CDD is the most obvious.

## Discussion and conclusion

### Theoretical contributions

The current concerns about UBC in education often only refer to activities in the education and research domain. Even from the perspective of HEIs, the valorization domain activities in UBC are of great significance. Commercialization and entrepreneurship activities can not only promote academics' research and open up a new view but also bring practical knowledge to students and provide innovative power and source for promoting the regional economy. Therefore, valorization activities are an inseparable part of the university mission. At the same time, to understand the driving factors of UBC, it is not enough to only measure individual motivations because environmental factors will directly affect the effect of behavior and also regulate the result by influencing motivation. Based on the theory of social cognition, this study also examines the influence of the internal mechanism and external environment of HEIs on UBC activities and its interaction with motivation and UBC activities as a moderator variable. The results show that motivation sets are different for different UBC activities, and the internal mechanism and external environment also have apparent differences in the regulation between motivation and UBC activities.

At the end of extrinsic motivation, money- and career-oriented motivations are significantly related to research and valorization domain activities, but not significantly related to education domain activities, especially those negatively significantly related to SD. However, education and social-oriented motivations at the end of intrinsic motivation are highly related to educational and valorization domain activities, and exceedingly, research-oriented motivation is significantly related to educational research and valorization domain activities. It can be seen that research orientation is the strongest of all motivations, which may be related to the current research pressure prevailing among Chinese academics. Research orientation motivation may exist in all career-related activities. In addition, education, research, and social orientation motivations are significantly related to the three types of activities, which is very reasonable because they correspond to the three missions of HEIs, respectively. The motivation of money and career orientation is only significantly related to research and valorization activities, but not educational activities, which is inconsistent with the previous view that engagement in UBC activities is mainly for extrinsic motivation (Di Nauta et al., 2018). Previous studies often focused on the relationship between UBC valorization activities and motivation without a comprehensive perspective. The conclusion of this study is of great significance for understanding UBC activities because it confirms that money and career orientation motivations only have a strong correlation between research and valorization activities, and the driving force of educational domain activities is more manifested as internal motivation.

The support mechanism shows the most substantial significance among the three elements of the internal mechanism, which is significantly related to the five activities. The supporting mechanism is essential to understanding, analyzing, and improving UBC (Galan-Muros and Davey, 2017). The effectiveness of support mechanisms in eliminating or reducing barriers or driving UBC has been widely recognized (Tetrevova and Vlckova, 2018). More interesting is that the management mechanism has a strong positive correlation with CDD, SD, and R&D and a strong negative correlation with LLL, PM, and COM. The content of the management mechanism includes communication, coordination, and setting basic standards for some activities. Therefore, it has two sides. It may promote some activities or hinder other activities because of the restrictions. The influence of the policy environment is the most obvious in the external environment. In China, UBC is usually a formal activity initiated by the government, so it is very reasonable to be strongly affected by policy factors. However, the economic environment significantly impacts PM, COM, and ENT and has no significant correlation with educational domain activities. It is because educational activities are not related to market factors. Regardless of the economic environment, the government guarantees and supports educational activities.

Another significant theoretical contribution of this study is demonstrating the regulatory relationship between environmental factors and motivation for UBC activities. The regulatory effects of different factors are different between motivations and UBC activities. In the internal mechanism and external environment as moderator variables, all factors have a positive regulatory relationship between motivation and UBC activities to varying degrees, which confirms the research hypothesis of this article. Among them, the support mechanism, policy environment, and management mechanism have noticeable positive regulating effects, consistent with the aforementioned conclusions, and further illustrate the importance of the support mechanism, policy environment, and management mechanism for driving UBC activities. Another theoretical contribution of this part is that the research conclusion also demonstrates the applicability of the social cognitive theory in such research. Environmental factors will also affect individual behavior by regulating individual motivation.

The regulating role is mainly concentrated in the four activities of CDD, PM, COM, and ENT. PM is bringing scientific research and technology to the market through the transaction of intellectual property assets, which is affected by the relevant mechanisms of schools and enterprises, and the local economy, policies, laws, and other factors. ENT refers to the activities of students and teachers to start enterprises in HEIs. PM refers to the flow of employees between colleges and enterprises for work. It can be seen that PM, COM, and ENT are directly related to HEIs, business, political, legal, and economic environments, and it is not surprising that they show a significant regulatory effect (Orazbayeva et al., 2019a). However, all factors have no significant correlation between LLL and motivation, which is consistent with the previous conclusion. Motivation and environmental factors have not significantly promoted the LLL activity level. The possible reason is that LLL occurs within the scope of continuing and distance education, which is not given the same attention as other UBC activities in Chinese universities. The respondents' engagement is not high, and the corresponding motivation level is also low.

### Managerial and policy implications

The practical significance of this article is that it provides a reference for policymakers and managers to formulate more effective plans for the development of UBC activities. The results show that the incentive conditions for different UBC activities should be different. For example, only providing external incentive conditions may not positively affect education-driven UBC activities. In addition, intrinsic motivation, especially education and research-oriented, can promote all activities. Therefore, policymakers and implementers should pay attention to the motivation in this aspect and strive to promote it. At the same time, in any case, we should ensure the integrity of the support mechanism in the pillar and strive to create a good policy environment, both of which have a good performance in both the direct incentive effect on UBC activities and the positive moderate motivation and UBC activities as moderator variables. In the conclusion of this article, the significant correlation between strategic mechanisms and UBC activities is not as apparent as other factors. The reason for the above results may be that the strategic mechanism cannot stimulate UBC activities, or the respondents do not understand the content of the strategic mechanism.

### Limitations and further research

This study also has limitations based on particular theoretical and practical value. First, the individual differences in the identity, age, gender, university experience, business experience, UBC experience, and teaching experience of the respondents will affect UBC activities. However, the explanation of the specific impact of these variables on the degree of UBC is beyond the scope of this article. In order to understand UBC more comprehensively, these factors need to be included in future research. In addition, this study only distributed questionnaires to universities in southwest China, and the results may differ in other regions. In future, it should be conducted in a broader range of regions. In order to have a deeper understanding of the factors driving UBC, qualitative research can also be added after quantitative analysis. Through interviews with respondents, we can have a deeper understanding of UBC.

## Data availability statement

The original contributions presented in the study are included in the article/Supplementary material, further inquiries can be directed to the corresponding author.

## Author contributions

HZ and XC: conceptualization, data collection, and data analysis. HZ, XC, YL, and ML: methodology, writing-original draft preparation, and writing-review and editing. All authors have read and agreed to the published version of the manuscript.

## Funding

This article is the research achievement of the Chengdu University Foundation (2022).

## Conflict of interest

The authors declare that the research was conducted in the absence of any commercial or financial relationships that could be construed as a potential conflict of interest.

## Publisher's note

All claims expressed in this article are solely those of the authors and do not necessarily represent those of their affiliated organizations, or those of the publisher, the editors and the reviewers. Any product that may be evaluated in this article, or claim that may be made by its manufacturer, is not guaranteed or endorsed by the publisher.
